# Standards-based metadata procedures for retrieving data for display or mining utilizing persistent (data-DOI) identifiers

**DOI:** 10.1186/s13321-015-0081-7

**Published:** 2015-08-08

**Authors:** Matthew J Harvey, Nicholas J Mason, Andrew McLean, Henry S Rzepa

**Affiliations:** High Performance Computing Service, Imperial College London, London, UK; Department of Chemistry, Imperial College London, South Kensington Campus, London, SW7 2AZ UK

**Keywords:** Metadata standards, DOI, Digital repository, Data retrieval

## Abstract

**Background:**

We describe three different procedures based on metadata standards for enabling automated retrieval of scientific data from digital repositories utilising the persistent identifier of the dataset with optional specification of the attributes of the data document such as filename or media type.

**Results:**

The procedures are demonstrated using the JSmol molecular visualizer as a component of a web page and Avogadro as a stand-alone modelling program. We compare our methods for automated retrieval of data from a standards-compliant data repository with those currently in operation for a selection of existing molecular databases and repositories.

**Conclusions:**

Our methods illustrate the importance of adopting a standards-based approach of using metadata declarations to increase access to and discoverability of repository-based data.

**Graphical abstract:**
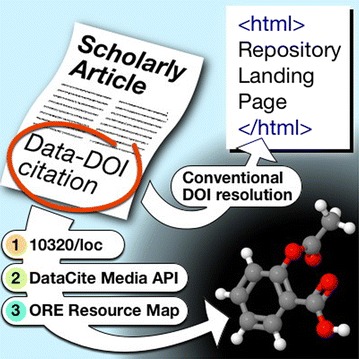
:

## Background

The scientific article as a component of a scientific journal has reached its 350th anniversary [1]. The structure that has emerged over that period is a combination of narrative and the data supporting that narrative, the latter often in the form of images and diagrams. The online journal era, dating from around 1996 onwards, has allowed a proportion of the visual and numerical data components to increasingly separate into what is often referred to as supporting information (SI or ESI for electronic forms) or other synonyms. Whilst the typical length of the narrative, still entwined with the more important data and visuals has in fact not changed much (~2–20 printed pages), in recent years the SI has ballooned in size. In areas such as synthetic chemistry, SI exceeding 100 pages expressed as a single monolithic PDF document is not unusual. The well-intentioned objective is to ensure that others can readily reproduce the experiments on which the narrative might have been based. Unfortunately, the data structures and other information contained in this super-sized SI remains largely uncontrolled by publishers, and one suspects only lightly inspected by referees if at all. There are few formal validation mechanisms for chemical data, a notable exception being tools for validating crystallographic data [2]. Metadata, data describing the data in the SI document, is rarely gathered. This in part is due to the lack of consistent syntactical and semantic standards for expressing it and often means that the resulting SI may only be processable by a human via visual inspection and interpretation.

In an earlier article on this theme [3], we argued a case for scientific articles to be cast as a datument, being a document in which the data within it is clearly identified in both a syntactical and a semantic manner, together with the associated narrative logical flow from which new knowledge can emerge. Our example used HTML mark-up to achieve a co-existence of these components, whilst retaining the narrative in a form more familiar not only to most readers but also the putative authors of the content. More recently we have adopted a refined model, recognising there are significant differences in how either narrative or data can be best expressed and published. This new model makes use of digital repositories as a more optimal medium for publishing data, whilst retaining the conventional journal article for the narrative. To achieve this model, we made use [4] of persistent identifiers such as the well-known DOI or digital object identifier for both the narrative and the data.

The use of the DOI to uniquely identify journal articles is now essentially universal amongst most publishers of scientific journals and is supported by the DOI registration agency CrossRef [5]. The CrossRef DOI infrastructures, which are now more than 10 years old, have inevitably been optimised for the purpose of linking to individual journal articles. Because most journals are in fact commercial activities, the infrastructure has evolved to recognise this feature; a DOI does not point to an article itself, but to what is called its landing page. This page, the layout and structure of which may vary according to the publisher, provides the mechanism for the reader to acquire the article itself. This in turn may require the reader to have access to an institutional subscription to the journal, or if this is not available, to provide a credit card to pay for the individual article. In some domains such as chemistry, only a small proportion of articles are currently placed outside of this paywall for unrestricted open access [6]. Crucially, the same landing page is used to provide links to the SI associated with the article, allowing the reader to download a PDF or other type of file containing the data. This link will probably but not invariably be outside the paywall. A small proportion of data might be provided in a more suitable structured form such as a CIF file [2] containing crystallographic information, but in general the media type [7] for the data is not declared and is not discoverable. Navigation of the landing page tends to be unique to each journal. There are no declared standards for automated discovery of information there and there cannot be any certainty that the navigational paths off the landing page will be static and not change if the landing page is redesigned. Access to the SI cannot be assumed to be persistent; the reader has to interpret each parochial landing page for the mechanism to acquire supporting information, then download and store it locally. Even at this stage, the data is often found in inappropriate containers such as the PDF format, one that was never designed for the purpose of managing data. Finally appropriate software to read and manipulate the data must be identified; this often reduces to the base level of a simple text editor, again a tool not necessarily optimised for extracting or manipulating data. There is rarely other supporting infrastructure to help the reader in this task; data is more than likely to be un-indexed, which means that searches for appropriate sub-components such as molecular connections or properties are unavailable. This lack of metadata means that the data may only eventually become discoverable via the traditional commercial abstracting agencies such as SciFinder, Reaxys or CCDC, where the expense of using humans to recover or curate the semantics and validation is reflected in the costs of the institutional licenses that are required to access that data.

In the last few years, some of these issues have started to be addressed by assigning persistent identifiers to data objects themselves. This has been expedited by the creation of agencies such as DataCite [8], who provide services and infrastructure to incorporate DOI registration into the functionality of digital repositories [9]. Whilst CrossRef was originally largely focused on journals, DataCite has from the outset concentrated on data repositories, albeit with a design for the infrastructure and recommended best practices that has been heavily influenced by those of the former. DataCite have developed unique data-oriented features and services such as the DataCite metadata schema which have been optimised for the act of data citation, following the first four articles of the Joint Declaration of Data Citation Principles (previously the Amsterdam Manifesto) [10]. These articulate the great value in the reuse of data [11] and recognise that since modern scientific data is often captured, interpreted and stored on machines, efficient reuse requires access that need not involve a human. It follows that the emerging best practice of using persistent identifiers (data-DOIs) to cite data within an article narrative or elsewhere necessitates a machine operable path from the persistent identifier to the data, particularly where data is made available on fully open terms. This concept can be succinctly summarised as DOI2Data. The DataCite services that have been developed for such content retrieval have so far not been significantly adopted for the purpose of archiving SI and most data repository landing pages still require human interpretation and navigation. In this article we describe working examples using three different metadata-based procedures that address these issues.

## Methods

Our first solution to the aforementioned problems utilized creating collections of data files on a Dspace-based repository. DSpace-SPECTRa [9] (Submission, Preservation and Exposure of Chemistry Teaching and Research Data) is a DSpace based repository that archives research data, primarily computational data generated using a high performance computing (HPC) resource. Each set of files (a fileset) in DSpace-SPECTRa tends to be associated with a discrete molecule, and each of these filesets is assigned a persistent identifier known as a handle, together with metadata describing the molecule and some of its properties [4]. Further metadata records were then made available to the handle manager to allow direct access to the data via a URL which can be derived from the handle, thus obviating the need to navigate a landing page.

The first method we implemented [4] that allows this procedure employed a standard known as 10320/loc [12]. The DataCite service does not currently support this standard, but they do provide two alternative methods based on a persistent DOI identifier assigned using their own registry. Before describing our solutions based on these methods, we recapitulate the essential features of the 10320/loc implementation based on handle records.

### Method 1: using features of the Handle System

The Handle System as maintained by CNRI [13] is in fact also the underlying technology behind the DOI system [14] noted above. Typically, the handle record of a DOI will consist of a URL value, to which the browser is redirected when the DOI is resolved by an agent such as the proxy servers http://doi.org/ or http://hdl.handle.net/. This URL normally points to a human-readable landing page. The 10320/loc specification is a handle value type that was introduced [15] to “improve the selection of specific resource URLs and to add features to the handle-to-URL resolution process”. This system requires these additional 10320/loc types to be added to the handle records via the handle manager, which in our case is a component of the DSpace-based SPECTRa digital repository system [4, 16]. The type consists of an xml-encoded list of file locations that can be filtered by attribute by appending a locatt parameter to the DOI-string requiring resolution. Examples are shown in Table 1 (entries 1–3).

**Table 1:** Different methods for using standards-based metadata to directly retrieve data files from a digital repository

Entry	URL or action	Function
1	http://doi.org/10042/32205?locatt=id:5	Using the locatt (location attribute) features to modify handle resolution based on the sequence identifier
2	http://doi.org/10042/32205?locatt=filename:MOPAC-PM7.out	Using the locatt (location attribute) features to modify handle resolution based on the filename
3	http://doi.org/10042/32205?locatt=mimetype:chemical/x-mopac-output	Using the locatt (location attribute) features to modify handle resolution based on the MIME type
4	onclick=“handle_jmol(‘10042/32205’)”	Modifying handle resolution by processing the handle response using a JavaScript event based on the handle_jmol script [28], with the default selection set to the media type: chemical/x-cml
5	http://data.datacite.org/chemical/x-cml/10.14469/ch/26199	Using the DataCite metadata store to specify a media type, allowing a direct URL to this data to be passed for display
6	onclick=“datacite_jmol(‘10.14469/ch/26199’)”	Displaying a list of available files using a JavaScript event based on the datacite_jmol script [28], allowing the user to select one
7	onclick=“datacite_jmol(‘10.14469/ch/26199?chemical/x-cml’)”	Selection by MIME-type with no user intervention using a JavaScript event based on the datacite_jmol script [28]
8	onclick=“datacite_jmol(‘10.14469/ch/26199?PM7.xml’)”	Selection by Filename with no user intervention using a JavaScript event based on the datacite_jmol script [28]
9	http://doi.org/10042/32205?noredirect	Determining the redirector for a specified DOI and the 10320/loc records (if any) for that entry
10	1. http://data.datacite.org/10.14469/ch/26199 (DSpace-SPECTRa) [9]	Determining the metadata records for a specified DOI
	2. http://data.datacite.org/10.6084/m9.figshare.1270384 (Figshare) [32]	
	3. http://data.datacite.org/10.5517/CC11TJ7M (CCDC) [35]	
	4. http://data.datacite.org/10.14272/XFNLWZCTEDTRGB-KJWHEZOQSA-N.1 (Chemotion) [39]	

Alternatively, since the 10320/loc locations are machine readable, the handle record can be retrieved using the handle REST API [15] and can be processed using JavaScript to return the URL of a specified file of interest (Table 1, entry 4). Currently, DataCite do not support the use of 10320/loc records through their client APIs. To address this lack, we have developed two further solutions.

### Method 2: using the DataCite content resolver and media API

The DataCite metadata store (MDS) API [17] includes a media resource, where MIME types can be associated with URLs as key:value pairs. Instead of redirecting to the usual landing page, a DOI can then resolve to these alternative URLs through content negotiation. This couples technology developed through Crosscite [18, 19] with the DataCite Content Resolver (Table 1, entries 5, 10). To make use of this feature, an application could resolve a given DOI, whilst specifying acceptable file formats as a list of MIME types in the Accept header of the HTTP request. For example, a request from an application that visualises chemical data might include a list of chemical-MIME types [7] such as chemical/x-cml. Resolution would then return a data file that matches one of requested formats, so long as that file has been registered against its type using the DataCite Media API. The Content Resolver also exposes URLs registered with the Media API through HTML links, as demonstrated in Table 1 (entry 5, for which metadata relating to the chemical/x-cml media type to enable this procedure has been added).

The DataCite content resolver has its limitations when a fileset has more than one file of the same MIME type. In this respect, it is less flexible that the 10320/loc**-**based solution previously described, where files may be selected not only based on MIME-type but also by filename (Table 1, entry 2), file ID (Table 1, entry 1) or any other specified attribute and for which content negotiation [20] is also possible. One way of avoiding conflicting MIME-types is to assign a DOI to each individual file, following the principles of functional granularity [21]. However, many data repositories, including DSpace-SPECTRa, allow multiple files to be associated with a single deposition and will assign these collections a single DOI, as is usually adequate for data citation. In DSpace SPECTRa, we have dealt with the issue of conflicting MIME types by simply nominating a single chemical/x-cml file to be registered for each dataset, containing data for multiple molecules if necessary. The advantage of this approach compared with “Method 1” is that it is fully implemented by DataCite.

### Method 3: OAI-ORE Resource Maps exposed through DataCite metadata

Some of the limitations of the DataCite Media API-resource and Content Resolver described in “Method 2” can be overcome by exposing the directory structure of the published fileset in a machine discoverable and readable way. ORE [22] (object reuse and exchange) is a standard maintained by the Open Archives Initiative (OAI) for describing aggregations of web resources through documents referred to as Resource Maps. These can be serialised in various formats, including Atom (used for RSS feeds), RDF or RDF-a (for declaring RDF triples). The DSpace repository server automatically creates an Atom OAI-ORE Resource Map for each repository object, as well as a METS [23] (metadata encoding and transmission standard) metadata file, which is an alternative standard that also provides the appropriate structural metadata. These files can themselves be made discoverable by including their locations as *related identifiers* within the DataCite metadata for the repository object. This utilises the *HasMetadata* relation type, introduced in version 3.0 of the DataCite schema [24]. Metadata is mapped internally from the Dublin Core schema used by DSpace to DataCite schema version 3.0, using a modified version of the DIM2DataCite.xsl XSLT transform (also known as a Crosswalk) originally designed for version 2.0 of the schema [25]. The DataCite metadata for a given assigned DOI can then be retrieved through content negotiation [20] and from this the ORE or METS metadata if found can then be retrieved and processed to return the URL of a specified file of interest. Exposing the URL of the OAI-ORE Resource Map through the DOI metadata also provides a solution to the more general issue of ORE Resource Map discoverability [22].

An advantage this approach has over our previous 10320/loc-based solution is the use of more widely adopted established standards. It makes use of OAI-ORE [22], a standard well designed for describing complex digital objects, here exposed through DataCite metadata [8], another de facto standard that is tightly coupled to the persistent identifier of the dataset. The flexibility of the approach makes it complimentary to that described in “Method 2” above. However, it is less efficient than the previous methods (Table 2), requiring several HTTP requests and xml processing of the returned responses, for which we have used JavaScript for our browser-based implementation. This however brings a disadvantage that code equivalent to this JavaScript functionality has to be written for each application that makes use of this procedure. A summary of the three methods is given in Table 2 outlining the essential features of each.

**Table 2:** A comparison of the essential features of Methods 1–3

	Method 1	Method 2	Method 3
Persistent identifer	handle	DOI (DataCite)	DOI (DataCite)
Resolution mechanism(s)	locatt query or xml-processing via script or content negotiation	Content negotiation	xml-processing via script
Number of HTTP GET requests for file retrieval	2	2^a^	3^a^
Metadata standards	10320/loc	–	DataCite schema, OAI-ORE, METS

^a^For requests made directly to http://data.datacite.org/. A request via http://doi.org/ requires an additional HTTP request.

## Results and implementations

**Figure 1:**
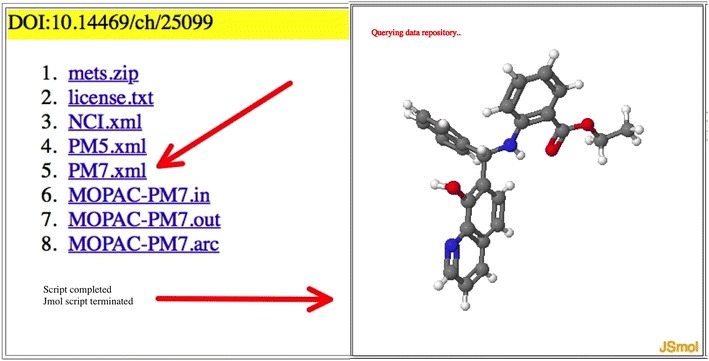
Data selection using OAI-ORE Resource Maps within the JSmol browser environment.

We have used two programs to illustrate these methods. The first is the molecular display program JSmol [26] we used to construct a demonstrator [27] for examples 1–8 (Table 1) and illustrated for example 6 in Figure 1. In each of these examples, only the persistent identifier (handle or DOI) for the data file need be specified, along with the media type of the file required as an option. This latter can be allowed to default to a specified type (Table 1, entry 4) to simplify the process. JSmol allows data to be loaded by specifying either a URL (Table 1, examples 1–3, 5) or by using custom JavaScript code [28] to pre-assemble a URL (Table 1, examples 4, 6–8) from the response obtained by querying the metadata associated with the persistent identifier for the dataset. Access to the data can be obtained using the JSmol program itself, which allows the visualized file to be saved to disk as a local file if required. Examples can also be found in a recently published article [4]. Examples 6–8 (Table 1) use the JavaScript function *datacite_jmol* [28], that implement “Method 3” described above and passes the results on to JSmol for visualisation. A list of available data files corresponding to the doi:10.14469/ch/25099 is shown in this example, and the reader can then select one of these for display.

**Figure 2:**
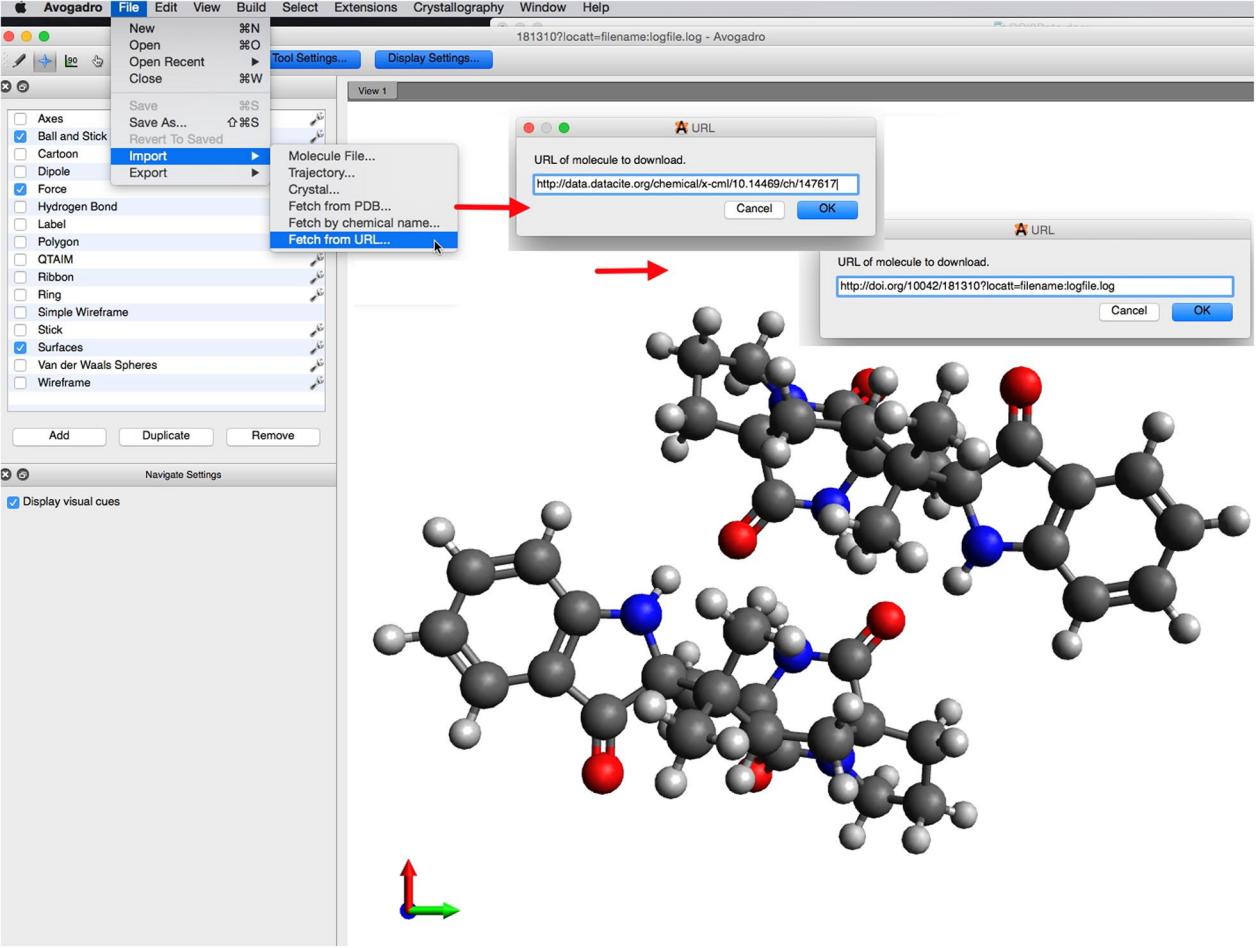
Visualisation of data using Avogadro by specifying a URL based on a persistent-identifier (DOI) using examples 2 or 5 (Table 1).

The Avogadro program [29] also supports importing molecular information by specifying a URL, in a manner similar to JSmol. The additional coding required to support this feature is minimal, requiring only that the program support URL redirection internally. A modified version of Avogadro can be used to access data using the syntax of examples 2 and 5 (Table 1) by specifying only the persistent identifier (DOI) together with information about the media type (Figure 2). Supporting the full-range of JavaScript-based procedures supported using the JSmol program would require further code additions to the current version of Avogadro.

## Discussion of alternative procedures used for data retrieval and display

One can in general envisage three broad levels of what might be called semantic information present in data associated with a declared persistent identifier.

- Coarsely grained data, which consists largely of a single data file or deposition described by a single set of metadata. An example of this model is a collection of 134 kilomolecules deposited in the form of a compressed archive comprising a single file and assigned one persistent identifier [30]. This model only has declared metadata for the entire collection and not for individual molecules in that collection; there are no molecular semantics declared via metadata for the components of this collection.
- Our model, in which each molecule is described by a data or fileset and has its own uniquely assigned persistent identifier. Some specific properties of the individual molecules described in the collection are semantically formalized, such as e.g. the InChI key or identifier. Most of the properties however have to be derived by the user after the data retrieval is complete by using additional tools.
- The most finely grained approach involves a more complete semantic ontology for the data, which allows more properties of the objects themselves to be formally declared. One evolving example of this approach is the collaboration between WikiData [31] and Wikipedia, where data exposed in the latter is transcluded by an appropriate semantic query of the former using a template such as ChemBox constructed for the purpose [31]. It will also be possible to access Wikidata via a public API, although is not yet apparent which metadata standards of the type discussed above will be supported.

Most well-known molecular repositories and databases currently adopt model (b) above, and we here compare the characteristics of a short selection of these with the three retrieval methods described above.

### Figshare

Figshare [32] is a relatively new architecture for digital repositories operating on a commercial basis, and one we have integrated into our own repository portal along with DSpace-SPECTRa [4, 16]. The redirection associated with the persistent identifier (DOI) of a typical deposition [33] can be obtained in a standard manner (Table 1, entry 9). This example returns the value: http://figshare.com/articles/Gaussian_Job_Archive_for_C3H6I2Zn/1270384 as the conventional landing page for the object. This page itself contains further direct links to individual files such as http://files.figshare.com/1842736/logfile.log or http://files.figshare.com/1842735/cml.xml. However, the path components 1842736 or 1842735 for these individual files are derived internally from the DOI by the Figshare system and are not available as declared metadata. A direct path can also be inferred from the landing page to the fileset bundle as http://downloads.figshare.com/article/public/1270384. In this instance it is related to the DOI, but again this information is not formally available via metadata and so cannot be assumed to be necessarily persistent. Whilst this latter link allows direct access, it is to the compressed archive 1270384.zip and the contents of this archive are again not declared in metadata; it would be necessary to download and expand this archive to acquire the information. So whilst direct paths from the DOI persistent identifier to Figshare data URLs can be inferred, they are not discoverable via metadata in a standard manner (Table 1, entry 10 and Figure 1). Semantic information in Figshare is expressed as tags, which are added manually during the deposition, or by using an API [16]. Thus a molecular entry may contain a tag such as an InChI identifier, but this property is not formally discoverable. This aspect is best illustrated by contrasting two DataCite DOIs, one minted for our SPECTRa repository and one created using Figshare:

1. http://data.datacite.org/10.14469/ch/26199 returns an Alternate identifiers attribute, one discovered value of which is the InChI key.
2. http://data.datacite.org/10.6084/m9.figshare.1270384 returns no such information.

### The Protein data bank [34]

This resource provides DOIs for individual entries assigned by CrossRef [5]. A typical entry with doi:10.2210/pdb1prc/pdb if resolved using the noredirect directive (Table 1, entry 9) shows a redirect to an FTP (file transfer protocol) server and a path to compressed archive of the dataset: ftp://wwpdb.org/pub/pdb/data/structures/divided/pdb/pr/pdb1prc.ent.gz. FTP is an older Internet standard with no mechanisms for specifying how metadata can be stored and hence queried. For example metadata about the contents of the file cannot be retrieved independently of the file itself. Nor is information that associates the file with the original assigned DOI declared explicitly on the FTP server itself. The PDB resource does provide a full API (application programmer interface) for access to data and much of its semantics, but this API is specific to this resource, and hence specific code has to be written to make use of it. Programs such as JSmol or Avogadro use hard-coded paths to the data rather than relying on dynamic information obtained from metadata-declarations. The request: http://data.datacite.org/10.2210/pdb1prc/pdb in this instance returns no information, since there is currently no exchange of information between CrossRef and DataCite.

### Cambridge crystallographic database centre (CCDC) [35]

In March 2014, CCDC began to associate a DOI with each individual entry using the DataCite API. If an example such as doi:10.5517/CC11TJ7M is invoked with a noredirect (Table 1, entry 9), this reveals it maps to the search engine interface: http://www.ccdc.cam.ac.uk/services/structure_request?id=doi:10.5517/cc11tj7m&amp;sid=DataCite which redirect to a HTML5-based landing page that also includes a JSmol [26] canvas, providing a visual rendering of the data. Access to that data can then be via the JSmol drop-down menu Show/file contents, producing a textbox containing the data that could then captured by a copy/paste operation. The metadata associated with the entry (Table 1, entry 10) shows no declared OAI-ORE or METS manifests or media types that could be used in any of the methods described above (Table 1, entries 5–8), but there is some declared semantic information associated e.g. with *Alternate identifiers* (the CCDC RefCode). These restricted options for data retrieval may be associated with the commercial nature of this database so as to prevent bulk mining of the contents.

### Pubchem [36]

In contrast to the CCDC system, Pubchem is an open database, potentially free of the types of restrictions shown in the previous example. However, there is no adoption yet of a persistent identifier to individual entries, each of which is defined by a simple (formally non-persistent) URL, as for example http://pubchem.ncbi.nlm.nih.gov/compound/2244 (the landing page for Aspirin). This page also has the synonym http://pubchem.ncbi.nlm.nih.gov/compound/aspirin. Although the site defines semantic metadata such as SMILES, InChI and InChIkey, this is not exposed in a harvestable standards-compliant manner (Table 1, entry 10). The data itself can be downloaded in a variety of syntactical forms using a download button, which invokes a generating script, but no metadata-based procedure derived purely from the compound identifier or CID (2,244 for this example) is available.

### Dryad [37]

This is a DSpace-based repository, and hence is related to the DSpace-SPECTRa system we describe above. Individual depositions are assigned a DOI using DataCite, and the metadata is similarly harvested (Table 1, entry 10). Programmatic access to the METS metadata manifest is fully documented [38] and is similar to that of “Method 3” outlined above. However, the key difference is that their six-step procedure begins with a query to Dryad’s OAI-PMH end-point, followed by retrieval of METS by insertion of the DOI into a URL template: http://datadryad.org/metadata/handle/INSERT_SHORT_ID_HERE/mets.xml. This procedure is not obviously discernible without first reading the documentation, or at least having knowledge of the underlying DSpace software and its methods. It could be made discoverable by including the location of the METS file in their DOI metadata, as explained in “Method 3”.

### Chemotion [38]

This is a new repository for both molecules and associated research data that uses DataCite to assign a DOI derived from the registered prefix and the InChI string of the molecule. Thus doi:10.14272/XFNLWZCTEDTRGB-KJWHEZOQSA-N.1 (Table 1, entry 10) redirects to http://www.chemotion.net/inchikey/XFNLWZCTEDTRGB-KJWHEZOQSA-N.1 and this is immediately followed by a second internal server redirection to the actual landing page for the data collection: http://www.chemotion.net/molecules/172.

The existence of individual data files for that entry is exposed using the DataCite *HasPart* relation type (Table 1, entry 10). This provides the individual datafile landing pages to information about the collection via further DOI *relatedIdentifiers*: http://dx.doi.org/10.14272/XFNLWZCTEDTRGB-KJWHEZOQSA-N/DEPT/90 and http://dx.doi.org/10.14272/XFNLWZCTEDTRGB-KJWHEZOQSA-N/DEPT/135 that themselves redirect to e.g. a further landing page: http://www.chemotion.net/inchikey/XFNLWZCTEDTRGB-KJWHEZOQSA-N/DEPT/135.

The actual data files themselves can only be accessed from these landing pages, taking the form: http://chemotion.s3-eu-west-1.amazonaws.com/datasets/2935/1D13C_DEPT135.jdx?AWSAccessKeyId=AKIAJMCBC2EPUMXQSXLQ&Signature=pxlxmpruWuIp4aCvOVZORVwzC3Q%3D&Expires=1427209008he.

This last URL that identifies the actual source of the data itself is not available via metadata and is probably not persistent.

It is important to note that whilst several of the above repositories have chosen to include meaningful identifiers or branding as part of their DOI strings, the DOI is formally an opaque identifier and the location to which it redirects may change in the future. Therefore, information cannot be reliably inferred from the identifier itself, and should instead be expressed through its metadata in a standard way [40]. There are numerous other databases of molecular information that we have not discussed. Our argument here is that even if such resources provide an API for access to the data held there, this requires specific code to be written for each instance. We argue that adoption of metadata-based standards based on persistent identifiers requires little or no additional code to be written to allow access to any specific instance. Adoption of the standards based persistent-identifier mechanisms discussed above would enable a more robust and easier way to provide such support.

## Conclusions

The examples discussed here in Table 1 have been created specifically to enable simple and automated retrieval of data from a standards-compliant data repository, with the purpose of bypassing the traditional landing pages of repositories associated with narrative articles (i.e. journals). An important benefit of adopting such a standards-based approach is that the data itself can be made much more discoverable using metadata declarations. Such infrastructures for handling data (SI) have been largely neglected in the traditional publishing models used in traditional journals, or instead incorporated into proprietary systems where the data ceases to be properly open. Our system in contrast remains open at all stages, and is particularly suited for the kind of high throughput retrieval that is required of data mining and related activities. It would surely be fitting to celebrate the 350th anniversary of the founding of the first scientific journal if the scientific research community were to agree to emancipate their data [41] by encouraging adoption of standards-based schemes such as the ones we describe here.
